# Reduced Antioxidant Response of the Fan Mussel *Pinna nobilis* Related to the Presence of *Haplosporidium pinnae*

**DOI:** 10.3390/pathogens9110932

**Published:** 2020-11-11

**Authors:** Antonio Box, Xavier Capó, Silvia Tejada, Gaetano Catanese, Amalia Grau, Salud Deudero, Antoni Sureda, José María Valencia

**Affiliations:** 1 Department of Agricultura, Ramaderia, Pesca, Caça i Cooperació Municipal, Consell Insular d’Eivissa, 07800 Balearic Islands, Spain; tonibox@conselldeivissa.es; 2 Instituto Español de Oceanografía, Centro Oceanográfico de Baleares, Muelle de Poniente s/n, 07015 Palma de Mallorca, Balearic Islands, Spain; xaviercapofiol@hotmail.com (X.C.); salud.deudero@ieo.es (S.D.); 3 Laboratory of Neurophysiology, Biology Department and Health Research Institute of Balearic Islands (IdisBa), University of the Balearic Islands, 07122 Palma de Mallorca, Balearic Islands, Spain; silvia.tejada@uib.es; 4 CIBER Fisiopatología de la Obesidad y Nutrición (CIBEROBN), Instituto de Salud Carlos III (ISCIII), 28029 Madrid, Spain; 5 Laboratorio de Investigaciones Marinas y Acuicultura, LIMIA-Govern de les Illes Balears, 07157 Port d’Andratx, Balearic Islands, Spain; gcatanese@dgpesca.caib.es (G.C.); amaliagrau@dgpesca.caib.es (A.G.); jmvalencia@dgpesca.caib.es (J.M.V.); 6 INAGEA (INIA-CAIB-UIB), Edifici Guillem Colom Casasnoves, 07122 Palma de Mallorca, Balearic Islands, Spain; 7 Research Group in Community Nutrition and Oxidative Stress and Health Research Institute of Balearic Islands (IdisBa), University of Balearic Islands, 07122 Palma de Mallorca, Balearic Islands, Spain

**Keywords:** *Pinna nobilis*, fan mussel, antioxidant defenses, oxidative stress, *Haplosporidium pinnae*, protozoan parasite, Balearic Islands

## Abstract

The endemic fan mussel (*Pinna nobilis*) in the Mediterranean Sea is at high risk of disappearance due to massive mortality events. The aim of the study was to evaluate the antioxidant response of *P. nobilis* collected in the Balearic Islands (Western Mediterranean) before and after the mass mortality event. Individuals collected before (between 2011 and 2012) and after (between 2016 and 2017) the event were analyzed by histological, molecular, and biochemical methods to compare pathogenic loads and biochemical responses. All the individuals collected during 2016–2017 presented symptoms of the disease and were positive for *Haplosporidium pinnae*, while acid-fast bacteria or/and Gram-negative bacteria were detected in some individuals of both sampling periods. The activities of the antioxidant enzymes catalase and superoxide dismutase in the gills were significantly lower in *P. nobilis* affected with the parasite compared to those in the asymptomatic ones, while levels of malondialdehyde, as an indicator of lipid peroxidation, were higher in infected individuals. When analyzing the differential effects of *H. pinnae* and *Mycobacterium* sp. on *P. nobilis*, it was observed that significant effects on biomarkers were only observed in the presence of *H. pinnae*. Co-infection of *P. nobilis* by *H. pinnae* with other pathogens such as *Mycobacterium* sp. constitutes a serious problem due to its high mortality rate in the Balearic Island waters. This concerning situation for *P. nobilis* is favored by a reduction in antioxidant defenses related to *H. pinnae* infection that induces oxidative stress and cell damage.

## 1. Introduction

The fan mussel *Pinna nobilis* (Linnaeus, 1758) is a Mediterranean endemic bivalve and one of the largest bivalves in the world, reaching up to 120 cm in length [1]. *P. nobilis* lives in coastal areas at a depth of 0.5−60 m, preferably in *Posidonia oceanica* and *Cymodocea nodosa* seagrass meadows [2]. During the last decades, *P. nobilis* populations have progressively decreased due to anthropogenic impacts, mainly related to the destruction of their habitats and anchorages [3,4]. This species was included in Annex II of the Barcelona Convention (1992) and in Annex IV of the EU Habitats Directive [5]. In Spain, *P. nobilis* was already considered as a vulnerable species in 2011 [6], but in 2016, a massive mortality event severely reduced populations to a few living individuals [4]. Due to the spread of these events of mass mortality along different areas of the Mediterranean Sea, *P. nobilis* was re-classified as “critically endangered” by the IUCN Red List of Threatened Species [7]. The mortality of *P. nobilis* in the Balearic Islands has mainly been associated with the presence of a haplosporidan protozoan parasite, identified as *Haplosporidium pinnae* sp. nov. [8]. Recently, other authors described a probable association with other pathogens to the mortality of the fan mussels, such as *Mycobacterium* sp. [9,10] or *Vibrio* sp. [11]. For example, in the Tyrrhenian coastline of Italy, a recent mass mortality of *P. nobilis* was directly associated with a mycobacterial disease [9]. Similarly, the analysis of moribund *P. nobilis* specimens from areas of Italy and Spain (Cataluña) evidenced that mainly *Mycobacterium* sp. and *H. pinnae* but also *Vibrio* sp. and *Perkinsus* sp. contribute to disease pathogenesis [10]. Affected *P. nobilis* specimens showed clinical signs of illness, though not specific, such as mantle retraction, gaping, slow closing, slow response to touch, and reopening of valves after a short time, resulting finally in the death of the individuals [4]. The mortality event reached 98−100% of individuals in the populations along the Spanish Mediterranean coast [12], and later it extended to other areas of Mediterranean Sea including the Aegean [13] and Adriatic Sea [14,15].

Protozoan parasites are habitual pathogens of several bivalves that occasionally cause massive mortality episodes [16,17]. In most of these cases, although the depletion of bivalve populations in general was massive, values close to 100% were not reached [18]. However, some occasions such as the iridoviral infection of *Crassostrea angulata* in the 1960s and the *Bonamia exitiosa* infection of *Crassostrea ariakensis* in the 2000s produced near total mortality [19,20]. Haplosporidan parasites have also been responsible for marine bivalve mass mortalities of oyster, mussel, and clam around the world [21]. In fact, although commercial exploitation of some of these species has collapsed in some areas as a consequence of pathogen infection, their extinction was never considered a realistic possibility [16,22]. In the same way, non-commercial bivalves *Spondylus gaederopus* and *Arca noae* were affected by die-offs in the Mediterranean Sea, but they showed reduced mortality percentages and/or affected extensions compared with the mortality of fan mussels [23,24].

In the presence of pathogens, organisms, including mollusks, produce and release important innate immune components, such as reactive oxygen species (ROS) and cytokines, to cope with infection [25,26]. The main cellular response in mollusk consists in phagocytosis, which involves the internalization of the pathogen by the fusion of the cell membrane around a microorganism and subsequent degradation through hydrolytic enzymes, such as esterases, and the production of ROS [27]. ROS can kill pathogens as well as act as a secondary messenger in cellular signaling pathways [28]. However, ROS overproduction as a consequence of infection can cause oxidative stress and cellular damage [29]. The high production of ROS is associated with an increase in lipid peroxidation, the oxidation of proteins and even DNA [30,31]. In this sense, a previous study has observed an increase in the production of ROS and malondialdehyde (MDA), a marker of lipid peroxidation, in the marine bivalve *Mimachlamys varia* infected by the protozoan parasite *Perkinsus mediterraneus* [26]. In order to protect against ROS overproduction, mollusks have a wide variety of antioxidant (enzymatic and non-enzymatic) mechanisms [29,32]. Among these, the most important antioxidant enzymes are catalase (CAT), which is able to convert hydrogen peroxide (H_2_O_2_) in water; superoxide dismutase (SOD), which eliminates the superoxide anion generating the H_2_O_2_; glutathione peroxidases (GPx), which detoxify H_2_O_2_ and lipid hydroperoxides oxidizing reduced glutathione (GSH); and glutathione reductase (GRd), which reduces glutathione disulfide (GSSG) to GSH, contributing to the maintenance of the cellular redox status [30,31,33].

Altogether, the aim of the present study was to carry out a comparative analysis using oxidative stress biomarkers in *P. nobilis* collected in the Balearic Islands (Western Mediterranean) before and after the first detection of the massive mortality event associated with the parasitic infection of *H. pinnae* and the possible contribution of other pathogens such as *Mycobacterium* sp. to this event.

## 2. Results

### 2.1. Histology and Molecular Biology Analyses

**Figure 1:**
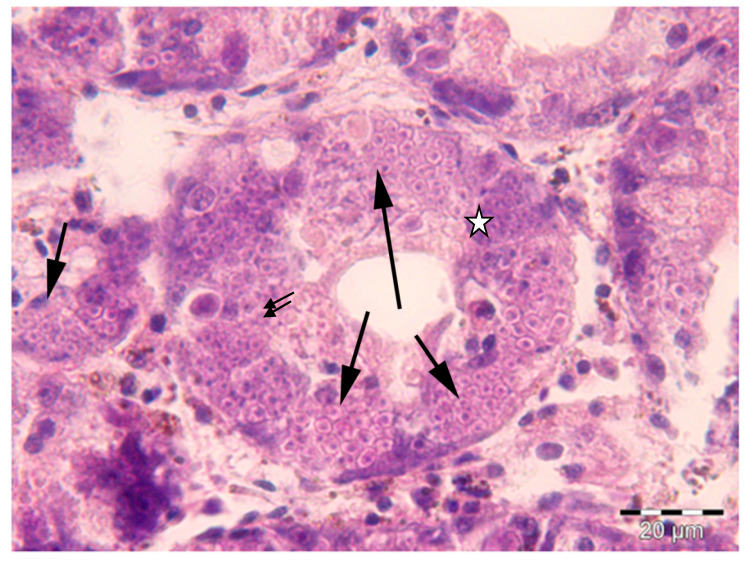
Histological section through the digestive gland of *Pinna nobilis* infected with *Haplosporidium pinnae* showing the epithelium of digestive gland tubules occupied by parasite sporocysts enclosing sporoblasts and more or less mature spores (**arrows**). Deeply stained plasmodia with cytoplasm compartmentalization are also seen (**star**) as well as free uninucleate cells (**double arrow**). Mayer’s hematoxylin and eosin (MHE) staining.

**Figure 2:**
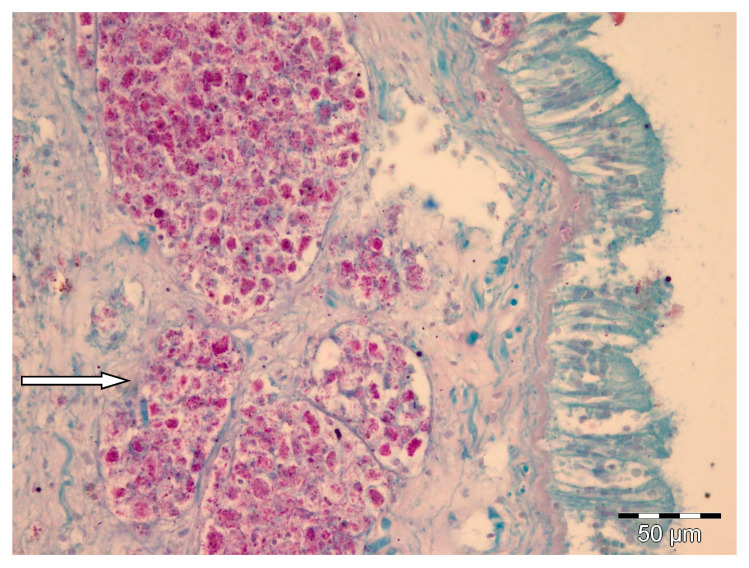
Histological sections through the digestive gland of *Pinna nobilis* with *Mycobacterium* sp. Nodular aggregates filled with acid-fast bacteria (**arrow**), Ziehl–Neelsen (ZN) staining.

The results of the histological and molecular biology analyses evidenced the absence of *H. pinnae* in the *P. nobilis* individuals collected between 2011 and 2012, when the mass mortality event had not been reported on the west coast of Mallorca yet. On the contrary, the presence of *H. pinnae* was observed in all the individuals collected in the same areas between 2016 and 2017 showing disease symptoms (Table 1, Figure 1). However, *Mycobacterium* sp. and Gram-negative bacteria were detected in both sampling periods (Table 1, Figure 2).

Amplicons of about 600 bp using the pair primers HPNF3/HPNR3 were obtained in all individual collected after the massive mortality event. PCR amplification using the primers mycgen-f/mycgen-r produced amplicons of about 1000 bp. The sequences of the PCR amplicons of the positive individuals for *H. pinnae* were subjected to BLAST analysis reporting 100% identity with a sequence present in GenBank (accession number: LC338065). BLAST analysis of the sequences from the positive individuals for *Mycobacterium* sp. failed to be identified to a level species, but they were very closely related, with 100% sequence similarity to *Mycobacterium* sp. identified in *P. nobilis* from the Tyrrhenian Sea (MH569645–MH569649).

All the individuals collected before the MME did not present *H. pinnae*, but this parasite was detected in all the specimens collected after the MME event. *Mycobacterium* sp. was present in 60% of samples collected before 2016 and in 50% of those samples collected before 2016. On the other hand, higher presence of other Gram-negative bacteria co-occurred in those individuals parasitized by *H. pinnae* (60%) compared to those that were not affected (40%), but mostly in coinfection with *Mycobacterium* sp. was prevalent.

**Figure 3:**
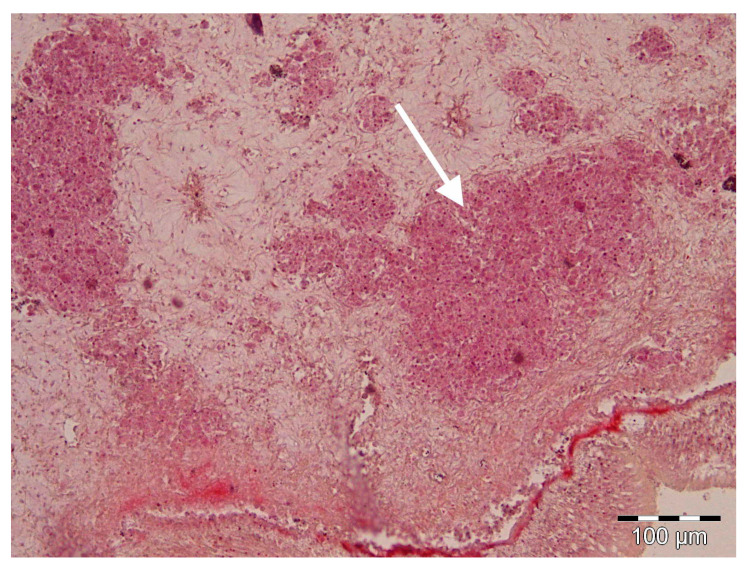
Histological sections through the digestive gland of *Pinna nobilis*. Nodular aggregates filled with Gram-negative bacteria (**arrow**), Gram staining.

Histologically, only after the massive mortality event, uninucleate cells of *H. pinnae* were observed in the connective tissue throughout all the visceral mass, as well as in the digestive gland epithelium, where different haplosporidan stages were also seen (Figure 1). On the contrary, in positive samples of both sampling periods, *Mycobacteria* were found inside nodular aggregates of immune cells located in the connective tissue surrounding the digestive gland (Figure 2). Gram-negative bacilli were detected in the same location (Figure 3).

### 2.2. Antioxidant Activities and Lipid Peroxidation Biomarkers

**Figure 4:**
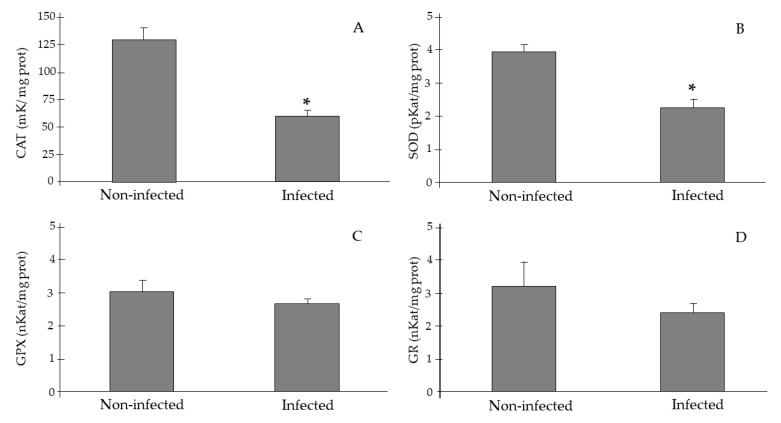
Antioxidant enzyme activities in gills of *Pinna nobilis* non-infected and infected by *Haplosporidium pinnae*. (**A**) Catalase (mK/mg prot), (**B**) superoxide dismutase (pKat/mg prot), (**C**) glutathione peroxidase (nKat/mg prot), (**D**) glutathione reductase (nKat/mg prot). * Indicates significant differences between infected and non-infected specimens (ANOVA *p* < 0.05).

Antioxidant enzyme activities in gills of *P. nobilis* are represented in Figure 4. The results showed a significant decrease in CAT (61.6 ± 3.9 vs. 129.2 ± 13.6 mK/mg prot, ANOVA *p* < 0.05) and SOD activities (2.29 ± 0.24 vs. 3.97 ± 0.19 pKat/mg prot, ANOVA *p* < 0.05) in *P. nobilis* infected by *H. pinnae* in comparison to non-infected individuals; whereas, no effects of *H. pinnae* infection in GPx and GRd were observed.

**Figure 5:**
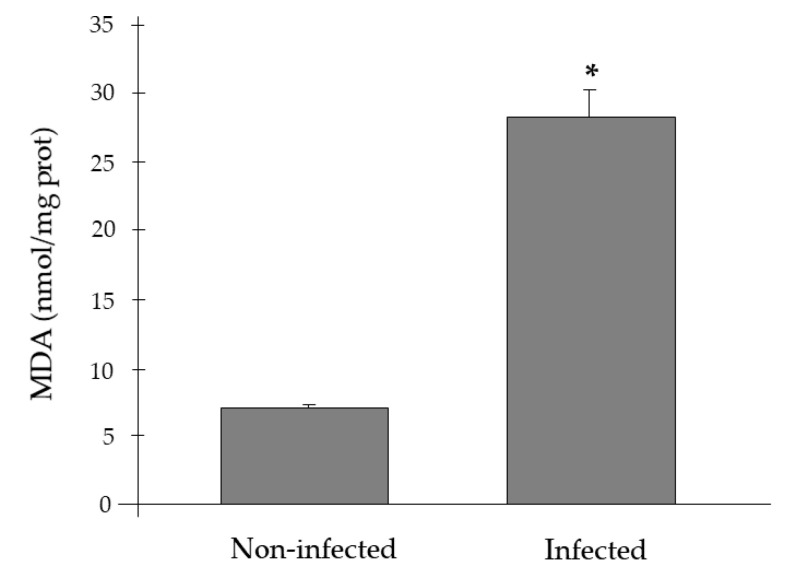
Malondialdehyde (MDA) levels (nmol/mg prot) in gills of *Pinna nobilis* non-infected and infected by *Haplosporidium pinnae*. * Indicates significant differences between infected and non-infected specimens (ANOVA *p* < 0.05).

Figure 5 represents the effects of *H. pinnae* infection on MDA levels. The results evidenced that the gills of *P. nobilis* infected by *H. pinnae* presented significantly higher MDA levels than those of non-infected individuals (28.26 ± 2.02 vs. 7.19 ± 0.30 nmol/mg prot, ANOVA *p* < 0.05).

The activities of the antioxidant enzymes and the level of MDA in the gills of *P. nobilis* separated according to the presence or absence of *H. pinnae* and *Mycobacterium* sp. are shown in Table 2. The results evidenced a significant decrease in catalase and SOD activities and an increase in MDA levels in the specimens of *P. nobilis* infected by *H. pinnae*, regardless of the presence or absence of *Mycobacterium* sp.

## 3. Discussion

The massive mortality event of *P. nobilis* that started in early autumn 2016 in the south-western Mediterranean Sea is a significant concern that has led to the loss of nearly 100% of the individuals in the Balearic Islands [4]. Since then, severe high mortality has also been observed in populations of *P. nobilis* from the north-western Mediterranean reaching the Aegean Sea [8,13]. Even recently, the Adriatic Sea has been affected by mass mortality events with mortalities between 36% and 100%, depending on the affected area [14]. This event involves the loss of an emblematic Mediterranean species; thus, monitoring plans, strict protection, and additional studies are a priority to mitigate the high risk of extinction and work toward the conservation of this species [34,35]. Although initially these massive mortality events were directly associated with the presence of *H. pinnae*, recent studies suggest that in some cases mortality is not linked exclusively to the infection by the protozoan, but that other pathogens such as *Mycobacterium* sp. or *Vibrio* sp. may also be responsible and, frequently, found co-infecting dead or moribund *P. nobilis* [9,10,36]

Healthy-appearing bivalves may have bacteria in their tissues, such as *Vibrio*, *Pseudomonas*, *Aeromonas*, and other Gram-negative forms frequently associated with adult bivalve mortality, although as secondary invaders rather than as primary pathogens [37]. Furthermore, the presence of acid-fast bacteria, *Mycobacterium* sp. (confirmed by molecular analyses), had been described in both healthy [14] and sick individuals of *Pinna nobilis* [9,10]. Healthy adult bivalves possess efficient humoral and cellular defense mechanisms acting against foreign material, destroying or eliminating it [38]. Histological analysis showed that the presence of a haplosporidan parasite was related to important lesions in the affected individuals of *P. nobilis* [8,39]. Moreover, the presence of different stages of sporulation of the protozoa [40] in the digestive gland confirmed *H*. *pinnae* as the main cause of the observed injuries [8]. The data provided by histological analysis, without the presence of *H. pinnae* in samples collected between 2011 and 2012 and with its presence in samples collected after 2016, were supported by molecular data; the presence of the parasite was confirmed by molecular analysis only in the infected *P. nobilis* presenting generic symptoms of the disease [8,41]. The fact of detecting Gram-negative and/or acid-fast bacteria in some individuals of both sampling periods suggests that although they can negatively affect *P. nobilis*, the differential factor and main cause of high mortality is *H pinnae*. In this sense, until the massive mortality event associated with *H. pinnae*, human stressors rather than environmental or other variables explained most of the variability in the spatial distribution of the density of *P. nobilis* in the Balearic Islands [42,43]. A previous work that analyzed the presence of the Gram-negative *Vibrio mediterranei* and other locally important pathogens of commercial bivalves in stabled *P. nobilis*, evidenced that *V. mediterranei* is an opportunistic bacterium that occurs in stressed *P. nobilis* individuals [11]. The authors also concluded that the presence of *Mycobacterium* sp. is not the cause of mortality in stabled *P. nobilis.* On the other hand, the results obtained in the present study showed a high presence of Gram-negative bacteria in those individuals affected by *H. pinnae* as well as by *Mycobacterium*, according to their opportunistic role in stressed specimens.

Marine bivalve mollusks have complex systems, which include humoral factors and cellular mechanisms, to defend against infection. Among the humoral factors involved in the elimination of pathogens, molecules such as antimicrobial peptides, major plasma proteins, lysosomal enzymes, lectins, and protease inhibitors can be considered [44,45]. The immune cellular response is mediated by hemocytes, which are responsible to phagocytize and eliminate invasive agents. Moreover, it is evidenced that hemocytes have an oxygen-dependent microbicide system, which eliminates pathogens using ROS [46]. It is also evidenced that ROS overproduction to eliminate pathogens can cause oxidative stress if they are not eliminated effectively [26,31]. Environmental adverse conditions such as pollutants or infections can induce oxidative stress in marine bivalves. In the presence of an infectious process, the hemocytes and the humoral factors of the mollusks act in a coordinated way to deal with the pathogens [44,45,46]. In addition to hemocytes, in the face of an infectious process, there is a generalized increase in cell metabolism causing an increase in ROS production, inducing the activation of antioxidant defenses. In this sense, a progressive increase in antioxidant enzymes was observed in the gills of bivalves (*Mimachlamys varia*) according to the degree of infection by the *Perkinsus mediterraneus* parasite [26]. However, the opposite was observed in the present study where a significant decrease of antioxidant defenses in individuals affected by *H. pinnae* was detected. This response might lead to the high mortality observed in *P. nobilis* affected by the parasite due to its inefficiency to respond to the infection.

Although the presence of *Mycobacterium* sp. and/or Gram-negative bacteria could have an effect on the antioxidant response, in this study the acid-fast bacterium was detected in individuals sampled both before and after the Spanish mass mortality event, thus not perturbing the total differences in the comparisons between the *H. pinnae* infected or not infected sample groups. Furthermore, when the response of the biomarkers to the presence/absence of *H. pinnae* and *Mycobacterium* sp. was differentially analyzed, significant changes were only observed in the presence of the protozoan. In fact, the results obtained in the present study contrast with those found in Campanian and Sicilian waters (Italy), where it is suggested that mycobacterial disease, and not *Haplosporidium* sp., is the main cause associated with mortality episodes of *P. nobilis* [9]. Moreover, it has already been reported that protozoans can reduce the host’s production of ROS facilitating their intracellular survival. The infection of *Ostrea edulis* with the protozoan parasite *Bonamia ostreae* in controlled conditions significantly reduced non-specific esterase activities, ROS production and the expression of extracellular SOD by hemocytes [27]. In addition, some bi-nucleated and tri-nucleated parasites were observed within the hemocytes suggesting that the parasite can divide inside hemocytes [47]. The ability of the parasite to divide could indicate that the protozoan’s own metabolic activity could contribute to ROS production and to oxidative stress.

The sporulation of the parasite in the digestive gland has been found to collapse the infected digestive cells, blocking the digestion process and leading to the death of the animal [8,39]. The inability to feed will not allow the affected animals to respond correctly to the infection and even significantly alter essential basic functions. Even, the lack of energy is evident by the almost null response to stimuli and slow closing of the valves [4]. The reduced eating capability will reduce the synthesis of new components that may contribute to the lower activity of antioxidant enzymes, favoring to increase the oxidative stress associated with *H. pinnae* infection itself. Accordingly, a previous report evidenced that oysters (*Crassostrea virginica*) infected by the protozoan *Haplosporidium nelsoni* presented a reduced condition index and fecundity compared to uninfected oysters, related to a reduction in the feeding rate [48]. The poor condition index suggests that chronically infected individuals would have depressed resistance to additional stress, favoring mortality events. In addition, total serum protein concentrations decreased in *C. virginica* in parallel to disease intensity suggesting that the depletion of certain metabolites and the disruption of biosynthetic pathways by the parasite may contribute to the death of infected oysters [49].

Together with the reduced antioxidant response, higher MDA levels were found in those individuals affected by *H. pinnae* indicating an increase in oxidative stress and damage to cellular components [50,51]. Although caloric restriction has been associated with a reduction in the degree of oxidative stress by reducing ROS production, if this situation is combined with other stressors such as metal exposure, or in this case to infection by *H. pinnae*, oxidative damage is notably increased [52]. In a mortality study on *C. virginica* larvae infected probably by *Vibrio* sp., a significant increase in MDA was observed in infected animals together with a reduced energy production that would prevent the adequate antioxidant response to efficiently resist the pathogen challenge [53]. Additionally, a decrease in SOD activity in the gills was observed in Manila clam *Venerupis* (=*Ruditapes*) *philippinarum* highly infected by *Perkinsus olseni* and also exposed to the toxic dinoflagellate *Alexandrium ostenfeldii* [54]. Moreover, a significant decrease in the activities of catalase, SOD, and GPx in the gills was evidenced in the shrimp *Palaemonetes argentinus* infected by the parasite *Probopyrus ringueleti* [55]. Since the lipid peroxidation process is a chain reaction with numerous bioactive intermediates, which could amplify and prolong oxidative stress induced by ROS, a decrease in the antioxidant capacity and in the ability to renew lipids damaged by the action of the pathogen will favor the appearance of end-products such as MDA [56]. Altogether, the mass mortality event that affects *P. nobilis* and the lack of antioxidant response suggest a collapse of the immune and antioxidant responses inducing oxidative damage and highly contributing to the mortality of the organism.

## 4. Conclusions

In conclusion, the infection of *P. nobilis* by *H. pinnae* is a serious problem in the Balearic Islands due to its high mortality rate, especially if it coexists with other infections such as *Mycobacterium* sp. or other Gram-negative bacteria, and has almost caused the extinction of the species throughout the entire Mediterranean Sea. In addition to blocking the digestive system, the infection by *H. pinnae* induced a collapse of the antioxidant defenses favoring the instauration of oxidative stress and cellular damage. This inability to activate the antioxidant system and increased oxidative damage can contribute to the high mortality related to the infection by this protozoan, especially if there is co-infection with other pathogens. Future studies under controlled conditions are necessary to clearly determine the involvement of *H. pinnae* and other agents such as *Mycobacterium* sp. in isolation or co-infecting on the massive mortality of *P. nobilis*. Finally, it is highly recommended to develop initiatives to preserve those *P. nobilis-*resistant populations or areas that are not infected by *H. pinnae* yet to conserve this Mediterranean emblematic bivalve species.

## 5. Materials and Methods

### 5.1. Sampling and Description of Collection Area

**Figure 6:**
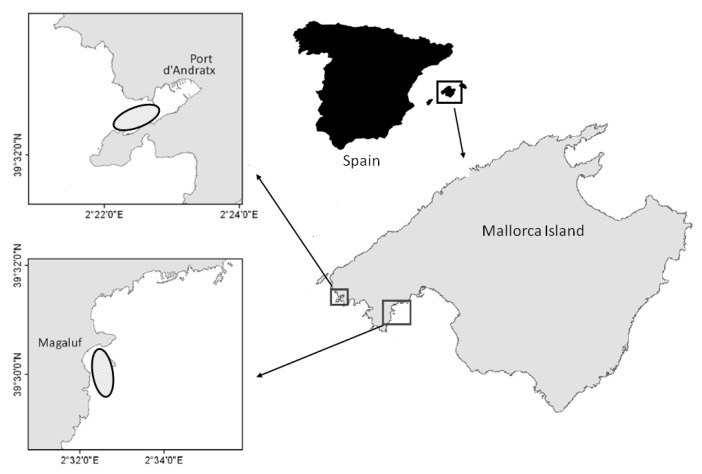
Map of Spain and Balearic Islands, indicating the sampling areas.

Due to the status of *P. nobilis* as an endangered and protected species, its sampling was carried out under the permission of regional and national authorities and collected by certified scuba divers at 8−10 m depth around Mallorca west coast (Magaluf and Port d’Andratx, Balearic Islands, Spain) (Figure 6). A total of 10 individuals collected before the mass mortality event (year of collection between 2011 and 2012) and 10 individuals collected between years 2016 and 2017 were used for histological, molecular, and biochemical analyses. All individuals collected between 2016 and 2017 presented generic symptoms of the disease (i.e., slow response to mechanic stimuli, keeping valves open, and slow valve closing).

### 5.2. Histological and Molecular Analyses

The sampled fan mussels were fixed in 10% phosphate-buffered formaldehyde or Davidson’s fixative for routine histological purposes [57]. A cross-section of the visceral mass of all the processed bivalves was taken at the level of the digestive gland, dehydrated in an increasing ethanol gradient, cleared with micro-clearing, embedded in paraffin wax, sectioned at 3−4 μM, and stained with Mayer’s hematoxylin and eosin (MHE) for routine light microscopic examination. Gram and Ziehl–Neelsen (ZN) staining was also performed to detect bacteria and acid-fast bacteria, respectively.

For molecular analyses, organs commonly infected by the parasite (digestive gland, adductor muscle, and mantle) were stored at −80 °C and then were dissected for DNA extraction. Total genomic DNA was purified using the DNA Tissue extraction kit (NucleoSpin^®^ Tissue, Macherey-Nagel, Germany) following the manufacturer’s instructions. To detect the presence of *H. pinnae*, fragments of the small subunit ribosomal DNA (SSU rDNA) gene were amplified using the pair primers HPNF3/HPNR3 [8], using the PCR reaction protocols previously described by López-Sanmartín et al. 2019 [41]. The presence of *Mycobacterium* sp. was detected by amplifying the 16S rRNA gene using the pair primers mycgen-f/mycgen-r described in Böddinghaus et al., 1990 [58]. PCR products were separated on 1% agarose in TAE 1× buffer gels (*w*/*v*), stained with GelRed^®^ Nucleic Acid Gel Stain (Biotium, CA, USA) including a LowRanger 100-bp DNA ladder size standard (NorgenBiotek, ON, Canada) and visualized on UV transilluminator. Obtained amplicons were purified using an mi-PCR purification Kit (Metabion International, Germany) following the manufacturer’s instructions and sequenced in a 3130xl DNA automated sequencer (Applied Biosystems, CA, USA). The sequences were aligned and edited using the BioEdit 7.1.3.0 software package [59] and compared using the basic local alignment search tool (BLAST) of the National Center for Biotechnology Information (NCBI).

### 5.3. Sample Homogenization for Biochemical Analyses

Gills of *P. nobilis* were homogenized in 10 volumes (*w*/*v*) of 100 mM Tris-HCl buffer pH 7.5. Each homogenate was briefly sonicated (2–3 s) using an ultrasonic processor and centrifuged at 9000× *g* at 4 °C for 10 min [30]. After centrifugation, supernatants were collected and immediately frozen and stored at −80 °C until analysis. All results were referred to the total protein content of the samples (Bio-Rad^®^ Protein Assay, CA, USA) using bovine serum albumin as standard.

### 5.4. Antioxidant Enzyme Activities

Enzymatic activities were measured in homogenate supernatants. Catalase activity (mK/mg protein; K = (s^−1^)) was measured using the method described by Aebi [60], based on the decomposition of H_2_O_2_. GPx activity was calculated using an adaptation of the method of Flohe and Gunzler [61]. Glutathione reductase (GRd) activity was measured by a modification of the Goldberg and Spooner’s spectrophotometric method [62]. Superoxide dismutase (SOD) (pmol/min/mg protein) activity was determined by the degree of inhibition of the reduction of cytochrome C by the superoxide anion generated by the xanthine oxidase/hypoxanthine system [63]. All antioxidant enzyme activities were determined with a Shimadzu UV-2100 spectrophotometer at 25 °C.

### 5.5. Malondialdehyde (MDA) Assay

MDA levels in the gills, as a lipid peroxidation marker, were analyzed in homogenate supernatants by a colorimetric assay for MDA determination based on the reaction of MDA with a chromogenic reagent to yield a stable chromophore with maximal absorbance at 586 nm (Merk, Madrid, Spain). Briefly, samples or standards were placed in glass tubes containing n-methyl-2- phenyl-indole (10.3 mM) in acetonitrile:methanol (3:1). HCl 12 N was added, and the samples were incubated for 1 h at 45 °C. Absorbance was measured at 586 nm.

### 5.6. Statistical Analysis

Statistical analyses were carried out using a statistical package (SPSS 21.0 for Windows^®^). The normal distribution of the data was assessed applying the Kolmogorov–Smirnov test. The statistical significance was compared by *t*-test for unpaired data to analyze the differences between *P. nobilis* infected with *H. pinnae* and those not infected. A one-way ANOVA followed by a least-significant-difference test was carried out to determine the statistical differences associated with the presence/absence of *H. pinnae* and *Mycobacterium* sp. Results are expressed as mean ± standard error of the mean (S.E.M.) and *p* < 0.05 was considered statistically significant.

## Figures and Tables

**Table 1:** Histological and molecular analysis results from *Pinna nobilis* samples collected around Mallorca Island before and after the massive mortality event. *Haplosporidium pinnae* and *Mycobacterium* detected by histological and molecular methods. Gram-negative bacteria were only detected by histology.

Date	*H. pinnae*	*Mycobacterium* sp.	Other Gram-Negative Bacteria	Date	*H. pinnae*	*Mycobacterium* sp.	Other Gram-Negative Bacteria
03/06/2011	−	+	+	24/11/2016	+	−	−
03/06/2011	−	−	+	24/11/2016	+	+	+
03/06/2011	−	−	−	24/11/2016	+	+	+
21/06/2011	−	+	+	03/03/2017	+	+	−
21/06/2011	−	+	−	03/03/2017	+	−	+
21/09/2011	−	+	−	03/03/2017	+	+	+
11/10/2011	−	−	−	03/03/2017	+	+	+
25/11/2011	−	−	−	03/03/2017	+	−	+
10/02/2012	−	+	−	26/06/2017	+	−	−
15/03/2012	−	+	+	26/06/2017	+	−	−

**Table 2:** Antioxidant enzymes activities and MDA levels in gill of *Pinna nobilis* depending on the presence or absence of *H. pinnae* and *Mycobacterium* sp. infection.

	*n*	Catalase	SOD	GPx	GRd	MDA
*H. pinnae* (−) *Mycobacterium* sp. (−)	4	115 ± 7	4.16 ± 0.43	3.24 ± 0.63	3.58 ± 1.25	7.10 ± 0.61
*H. pinnae* (−) *Mycobacterium* sp. (+)	6	138 ± 15	3.86 ± 0.10	2.98 ± 0.27	3.04 ± 0.72	7.13 ± 0.27
*H. pinnae* (+) *Mycobacterium* sp. (−)	5	62.0 ± 3.2 *	2.06 ± 0.29 *	2.68 ± 0.19	2.22 ± 0.19	28.3 ± 3.1 *
*H. pinnae* (+) *Mycobacterium* sp. (+)	5	61.8 ± 6.8 *	2.52 ± 0.32 *	2.73 ± 0.19	2.60 ± 0.48	28.2 ± 2.2 *

SOD, superoxide dismutase; GPx, glutathione peroxidase; GRd, glutathione reductase. One-way ANOVA, *p* < 0.05. * Indicates significant differences with respect to the groups with *H. pinnae* (−). Values are expressed as mean ± S.E.M.
